# Male Human Papillomavirus Infection and Genotyping in Turkey

**DOI:** 10.31557/APJCP.2023.24.12.4187

**Published:** 2023

**Authors:** Nilgun Tekkesin, Safak Goktas, Veysi Alkis, Efe Tekkesin, Pasa Goktas

**Affiliations:** 1 *Department of Clinical Biochemistry, Uskudar University, Istanbul, Turkey. *; 2 *School of Medicine, Maltepe University, Istanbul, Turkey. *; 3 *Gelisim Laboratories Molecular Technician, Istanbul, Turkey. *; 4 *School of Medicine, Istinye University, Istanbul, Turkey. *; 5 *Director of Special Gelisim Laboratories, Istanbul, Turkey. *

**Keywords:** Human papillomavirus, Genotype, Men, HPV infection, HPV DNA, High-Risk HPV

## Abstract

**Background:**

High-risk (HR) Human Papillomavirus (HPV) has been shown to play an important role in men in various locations in Turkey. This study aims to screen the male persistent infection with the high-risk human papillomavirus (HPV) genotype status in Turkey to provide a reference basis for formulating prevention strategies for the development of genitourinary tract neoplasia.

**Methods:**

The HPV QUANT-21 Quantitative RT-PCR Kit® was used to identify and quantify low-risk HPV (HPV 6, 11, 44) and high-risk (HPV 16, 18, 26, 31, 33, 35, 39, 45, 51, 52, 53, 56, 58, 59, 66, 68, 73, 82) from male individuals in Turkey. Results: Of the total 1304 samples, 473 were positive for at least one HPV genotype, with an overall frequency of 36.2%. Two-hundred fifty-four patients were positive only for one or more LR HPV genotypes (54%), and 219 patients were positive for one or more HR HPV genotypes (46%). The LR HPV genotype frequency was 53.7%, while the HR HPV genotype frequency was 46.3%. Our technology had the positive advantage of being able to calculate concentrations for each genotype. Genotype 51 was second in frequency but had the highest average concentration of 5.38 log (copies/sample).

**Conclusion:**

The presence and genotype of the virus before HPV vaccination are also of increasing importance. The data obtained will serve as a guide for prevention strategies, especially vaccination. Based on our findings there is a need of new estimates of the efficacy of currently available HPV vaccines and to develop a screening program to prevent and reduce the incidence of genitourinary tract neoplasias in Turkey. Further studies are planned to measure and define the high levels of infection that may lead to the development of cervical tumors. Using this technique, it may be possible to make clinical decisions about the extent of cytological alterations.

## Introduction

Human papillomavirus (HPV) is a Papillomaviridae family of a circular double-strand DNA virus (Lefkowitz et al., 2018). It is one of the most prevalent sexually transmitted viruses in men and women globally, and it can cause a wide range of dermatological issues and disorders, from benign warts to various kinds of cancer (Braaten et al., 2008; Liu et al., 2019). So far, over 230 different kinds of HPV have been identified (Schottenfeld et al., 2005; Else et al., 2011). More than 40 of these can infect the genitourinary tract. According to the International Agency for Research on Cancer (IARC), HPV is classified as low-risk or high-risk based on its carcinogenic potential. High-risk kinds are capable of integrating into the host DNA. In women, repeated or chronic infection with high-risk HPV is a required condition and the primary risk factor for cervical cancer. The low-risk variety is primarily associated with genital warts (Harden et al., 2017). Male HPV infection has been linked to an increased risk of cervical cancer in their sexual partners (Fogarty, 2007). However, the fact that HPV infection in men might be associated with significant morbidity is frequently ignored. Comprehensive studies of HPV infection and genotypes in men are relatively rare. To better understand male HPV infection and genotypes, we investigated male human papillomavirus (HPV) infection status and genotyping in Turkey. 

Furthermore, regional data on HPV prevalence and genotypic distribution are required to estimate the impact of vaccines on screening programs. Because, as previously stated, there is a causal association between HPV and several dangerous illnesses. HPV immunity is genotype-specific and knowing the incidence of LR- and HR-HPV in distinct geographic locations is crucial. The current study attempted to investigate the relative frequency and distribution of HPV genotypes in multiple genital samples acquired from males undergoing standard urological care examinations in different regions of Turkey. The frequency of genotypes other than HPV 16 and 18 in men is still unknown in Turkey. Furthermore, the measurement of the viral genome is a key value in making a critical decision. Knowing the frequency and diversity of genotypes will shed light on the effectiveness of the treatment and vaccination programs to be applied.

## Materials and Methods

*Urethral sample collection*

Our study consisted of an unknown open population, and the collection of the samples was conducted from January 2021 to May 2022 from all over the country. Open-population samples were obtained from men seeking routine urological care at several special healthcare centers or government hospitals and sent to a special laboratory as ordered to analyze HPV genotyping from all over the country. The open-population group was randomly selected without the knowledge of complaining about visual genital warts, history of HPV, being vaccinated before, and taking a known treatment therapy. We have no detailed information about the patient examinations or findings that all samples were included in the data except cases with insufficient data (without age) or poor DNA quality or quantity were excluded. A total of 1304 urethral brushings were obtained according to medical indication, from men aged in the range of 17-74 years old (years) (median 34 years) and consecutively enrolled in the study.

Urethral epithelium, anus, or penile epidermis samples were collected during an examination by physicians. The sampling using a device for self-sampling is carried out by the instructions for the use of special sterile disposable urogenital swabs. Before the sampling, it is necessary to remove the mucus with a cotton tampon and treat the area with a sterile physiological solution. The sampling swab is inserted into the urethral canal to a depth of 0.5 cm. 

Procedural limitations: local application of medicine, sexual activity less than 24 hours before the procedure. Men must not perform hygiene procedures or syringes before the sampling procedure. 

*Transportation and Storage of the Samples*

In the case of usage of transport media biological material samples were transported and stored according to the instructions for the transport medium used intended for subsequent sample analysis by PCR. Daily, samples were transferred to the microbiology laboratory in STOR-F transport medium (DNA-Tecnology, Russia). Samples should be stored at temperatures ranging from 2 to 4°C for no more than 24 hours. When it was impossible to deliver the material in the laboratory during the day, a one-time freezing of the material stored at temperatures of minus 20°C for one month was allowed.

*DNA Extraction*

DNA extraction was performed following the methodology recommended by the manufacturer using a commercial kit (Prep-NA Plus, DNA-Tecnology, Russia). 

*HPV Genotype Detection and Quantification*

Open-population samples were analyzed by a device named DTlite real-time PCR (DNA-Technology, Russia). HPV genotyping was done by HPV QUANT-21 Quantitative REAL-TIME PCR Kit® (DNA-Tecnology, Russia), which is intended for the specific identification and quantification of low-risk (HPV 6, 11, 44) and high-risk (HPV 16, 18, 26, 31, 33, 35, 39, 45, 51, 52, 53, 56, 58, 59, 66, 68, 73, 82) about their oncogenic properties of HPV. In the samples containing HPV DNA (specific product), the absolute quantity of this virus type was given as log (copies/sample) (the degree of concentration common logarithm, number of copies of the HPV DNA per sample).

*Ethics Committee*

There was no need to obtain informed consent from participants as the data was retrieved from daily HPV genotyping routines. All samples were analyzed in a special reference laboratory, named Special Gelisim Laboratories, located in Istanbul, Turkey. Before data collection, the laboratory requested a written confirmation report confirming that all participant data was handled confidentially. This study was approved by the Ethics and Research Committees of Nisantasi University, School of Medicine.

*Statistical Analysis*

Data were analyzed using IBM SPSS Statistics version 22.0 software (IBM Corp., Armonk, NY). Where applicable, data were expressed as median (interquartile range, IQR) and mean (standard deviation). Comparisons between groups were performed using the chi-square test or Fisher’s exact test. A statistically significant p-value of 0.05 was considered (two-tailed test).

## Results

A total of 1304 male subjects seeking routine urological care clinics of different hospitals in Turkey were recruited. From the total samples, 473 were positive for at least one HPV genotype, with an overall frequency of 36.2%. The other part of the samples from 831 men was HPV negative (63.8%). 254 patients were positive for only one or more LR-HPV genotypes (Genotypes 6, 11, and 44) (relative frequency of 54%) and 219 patients were positive for one or more HR-HPV genotypes (a relative frequency of 46%).

The participants ranged from 17 to 74 years and were analyzed in 4 different groups to evaluate the age distribution of the HPV positivity and negativity. The mean age of HPV-negative population was 44 years, whereas the HPV-positive population was 33.51 years, ending in age 74^th^. According to the four age groups, HPV positivity rates were: < 31 years (37.6%), 31-40 years (32.1%), 41-50 years (12.4%), and over 51 years (17.7%) (Table 1). Men aged under 31 years showed the highest overall HPV positivity with a rate of 37.6%. Comparing the presence of HPV genotypes in different age groups as statistically significant with p values of 0.000.

Although 259 samples (54.7%) were identified as a single HPV genotype, more than one HPV genotype was detected in 214 samples (45.3%). The age distribution in single and multiple genotypes was significant, as shown in Table 2. Multiple distributions was mostly detected between 31-40 ages, whereas single one was below age 31.

**Figure 1:**
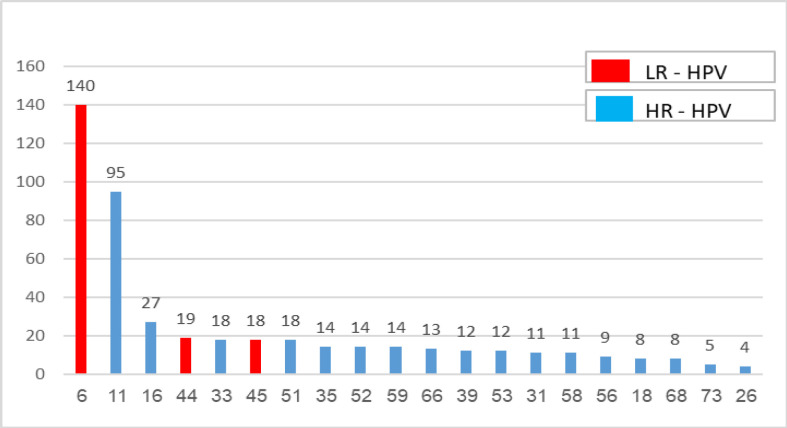
All HPV Genotypes Ordered in Frequency from Highest to Lowest Count

Twenty-one HPV genotypes ordered from the highest to lowest were given as frequency in Figure 1. While the frequency of LR-HPV genotypes was 53.7%, the frequency of HR-HPV genotypes was 46.3%. In the HR-HPV group, genotype 16 was at the top and genotype 82 was at the bottom. The most common 3 HR-HPV genotypes were HPV 16, with 27 counts (relative frequency of 5.7%), followed by HPV 51/45/33, with each 18 counts (relative frequency of 3.8%), and HPV 59/52/35 with each 14 counts (relative frequency of 2.9%) (Table 3). Those seven genotypes represented one-quarter of the most frequent HPV HR-positive genotypes (26%). HPV 26 and HPV 82 were the least seen genotypes, with 4 and 3 cases, respectively. The most common LR-HPV genotype was HPV 6, with 140 cases (relative frequency of 29.6%).

Age distribution percentages according to four groups for each HPV genotype were given in Table 4. Significant differences were observed for genotypes 16 and 56 (p=0,000).

As the concentration was calculated and given as a log (copies/sample), the mean values for each genotype were shown in Table 5. Although genotype 51 ranked second in frequency, it showed the highest mean concentration, with a 5.97 log (copies/sample) value. The total mean value for LR and HR genotypes was highly different as, 2.80 vs 4.47 log (copies/sample) (SD 0.09 and 1.22, respectively) (Table 6). The concentrations of LR-HPV and HR-HPV were mostly accumulated below 3 log (copies/sample) (p=0.000).

**Table 1:** Frequency of HPV-Positive or HPV-Negative Samples According to Age Groups and Presence of One or Multiple Genotypes

Age Groups (years)	HPV (+) numbers (%)	HPV (-) numbers (%)	p value
< 31	178 (37.6)	148 (17.8)	0
31 - 40	152 (32.1)	453 (54.5)	0
41 - 50	59 (12,4)	190 (22.8)	0
> 51	84 (17.7)	40 (4.8)	0

**Table 2:** HPV Positivity Rate in One and Multiple Genotypes

Age Groups (years)	Single. numbers (%)	Multiple. numbers (%)	p value
< 31	59 (22.7)	158 (73,8)	0
31 - 40	124 (47.8)	48 (22,4)	0
41 - 50	28 (10.8)	5 (2.3)	0
> 51	48 (18.5)	3 (1.4)	0

**Table 3:** Frequency of Low Risk-HPV and High Risk-HPV Genotypes from 473 Positive Men

HPV genotypes	n	%
LR-HPV		
Type 6	140	29.60
Type 11	95	20.08
Type 44	19	4.02
HR-HPV		
Type 16	27	5.71
Type 18	8	1.69
Type 26	4	0.85
Type 31	11	2.33
Type 33	18	3.81
Type 35	14	2.96
Type 39	12	2.54
Type 45	18	3.81
Type 51	18	3.81
Type 52	14	2.96
Type 53	12	2.54
Type 56	9	1.90
Type 58	11	2.33
Type 59	14	2.96
Type 66	13	2.75
Type 68	8	2.69
Type 73	5	1.06
Type 82	3	0.63

**Table 4:** Distribution of HPV Genotypes According to Age Groups Given as %

Age Groups (years)	HPV genotypes in %																				
	6	11	16	18	26	31	33	35	39	44	45	51	52	53	56	58	59	66	68	73	82
< 31	51.1	46.8	54.9*	40.5	49.7	45.5	51.1	43.7	46.8	49.6	40.5	51.2	42.8	47.1	50.1*	51.0	46.4	49.5	54.5	42.2	50
31 - 40	24.4	31.8	26*	32.3	32.3	32.4	31.7	36.3	30.4	33	37.8	33.9	39.8	33.1	33.8*	30.2	34.5	33.8	29.1	33.1	30.7
41 - 50	18.6	13.5	16.3*	18.3	14.3	13	12.4	12.5	16.1	15.1	18.9	10.2	13.9	15.1	12*	13.4	14.4	13.8	10.9	15.1	12.7
> 51	5.9	7.9	2.8*	4.4	3.7	6.7	4.8	7.5	4.8	4.7	2.8	4.7	3.5	4.7	4.1	5.4	4.7	2.9	5.5	9.6	6.6

*p value statistically significant

**Table 5:** Frequency of Low Risk-HPV and High Risk-HPV Genotypes

HPV genotypes	n	*Mean Value (SD)	*Minimum-Maximum Values
LR-HPV			
Type 6	140	2.69 (1.47)	0.4 - 4.9
Type 11	95	1.92 (1.52)	0.4 - 7.1
Type 44	19	3.79 (1.37)	0.4 - 9.2
Total	254	2.80 (0.09)	0.3 - 9.2
HR-HPV			
Type 16	27	5.35 (1.73)	0.93 - 11.3
Type 18	8	5.12 (2.21)	0.7 - 10.4
Type 26	4	2.71 (0.99)	1.6 - 11.6
Type 31	11	4.10 (2.31)	0.3 - 10.2
Type 33	18	4.40 (1,13)	1 - 9.8
Type 35	14	4.20 (1.24)	1.14 - 11.9
Type 39	12	5.20 (1.62)	0.5 - 12.6
Type 45	18	5.01 (1.99)	0.6 - 10.6
Type 51	18	5.97 (1.66)	1.7 - 11.7
Type 52	14	4.28 (1.72)	1.01 - 9.9
Type 53	12	4.99 (1.71)	0.3 - 12.6
Type 56	9	3.78 (1.69)	1.2 - 10.3
Type 58	11	5.70 (2.40)	2.3 - 9.4
Type 59	14	4.69 (2.01)	1.30 - 10.3
Type 66	13	3.76 (1.41)	1.63 - 10.5
Type 68	8	3.14 (1.22)	1.12 - 10.5
Type 73	5	4.12 (2.21)	1.4 - 10.6
Type 82	3	4.02 (2.13)	1.5 - 9.5
Total	219	4.47 (1.22)	0.2 - 12.3

*log (copies/sample)

**Table 6:** Distribution of LR-HPV and HR-HPV Concentrations

*HPV concentration ranges	n	*Mean Values (SD)	p value
LR-HPV			
<3.0	134	3.49 (0.31)	0
3.1-6.0	104	6.20 (0.7)	0
>6.1	16	2.70 (0.90)	0
HR-HPV			
<3.0	124	3.54 (0.21)	0
3.1-6.0	79	5.46 (0.54)	0
>6.1	16	2.61 (1.30)	0

*log (copies/sample)

## Discussion

Human Papillomavirus (HPV) is closely linked to several diseases and is one of the most common DNA oncoviruses frequently identified in clinics, nowadays. Furthermore, HPV infection is linked to 5.5% of malignancies worldwide (Schottenfeld et al., 2005). Also, HPV infection is one of the most common sexually transmitted illnesses, notably in the skin and anal genitalia. Therefore, early screening, timely treatment, as well as expanding vaccination coverage, are the main goals to improve the current situation. Determination of the type-specific HPV prevalence and distribution in specific areas are important parts of formulating prevention and control strategies to reduce the incidence of malignancies. The HPV QUANT-21 Quantitative Real Time-PCR Kit is an in vitro DNA test, which is intended for the specific identification and quantification of low-risk (HPV 6, 11, 44) and high-risk (HPV 16, 18, 26, 31, 33, 35, 39, 45, 51, 52, 53, 56, 58, 59, 66, 68, 73, 82) about their oncogenic properties of HPV. Therefore, in this study, we divided the HPV-positive samples into LR and HR HPV genotypes according to manufacturer recommendations. This method has the benefits of high accuracy, specificity, and sensitivity. This has been frequently employed in the identification of HPV infection by typing (Tekkesin et al., 2022). Before, we analyzed the women population in Turkey using this method with a high sample volume and commented on the HPV vaccination program (Tekkesin et al., 2022). Since this study included patients from both the health examination center and urology clinics, it is a more representative reflection of the overall HPV infection situation.

According to an HPV epidemiological study carried out among the general population in Turkey (nearly 85 million), the HPV burden among men in Turkey is mentioned (Denny et al., 2012). The information to date regarding anogenital HPV infection is primarily derived from cross-sectional studies of selected populations such as the general population, university students, and military recruits, and studies that examined husbands of control women, as well as from prospective studies in Turkey. Special subgroups include mainly studies that examined STD (sexually transmitted diseases) clinic attendees, MSM (men who have sex with men), HIV-positive men, and partners of women with HPV lesions, CIN (cervical intraepithelial neoplasia), cervical cancer or cervical carcinoma in situ. Globally, the prevalence of external genital HPV infection in men is higher than cervical HPV infection in women, but persistence is less likely. As with genital HPV prevalence, high numbers of sexual partners increase the acquisition of oncogenic HPV infections (Rodríguez-Álvarez et al., 2018).

HPV DNA is detectable in approximately 51% of all penile cancers (de Martel et al., 2020). Among HPV-related penile tumors, HPV16 is the most common type detected, followed by HPV18 and HPV types 6/11 (Miralles-Guri et al., 2009). In our study, LR-HPV was highly prevalent, with 54% of men testing positive for LR-HPV. Moreover, the present study showed that of overall HR-HPV prevalence, the frequency of other HR HPV, HPV16, and HPV18 were 46, 5,71, and 1,69%, respectively. On the other hand, Salehi-Vaziri et al., in 2015 reported a prevalence of HR-HPV in 9.5% of Iranian men referred to the Sexually Transmitted Infections 8(STI) clinics showing a prevalence of HR HP16, HPV16 and HPV52 were 2.3, 1.9 and 1.9% (Salehi-Vaziri , 2015). Those values were nearly similar to or higher than the prevalence in Iranian women (3.2%, 1.4%, and 0.9%, respectively) (Chalabiani et al., 2017).

Among the 473 HPV-infected subjects, the single HPV-type infection was dominant (57.4%) and significantly higher than that of the multiple infections (45.3%). Studies have shown that the existence of multiple HPV genotypes may prolong the duration of HPV infection and may increase the risk of cancer (Schmitt et al., 2013). Therefore, multiple infections are more dangerous than single infections. In this study, multiple forms were distributed mostly below age 31, suggesting an active and maybe uncontrolled sexual life has a threatening effect. However, studies have also shown that there may be competition or counterbalance between various types of HPV (Bernard et al., 2013).

Infection with multiple HPV subtypes would increase the risk of abnormal proliferation and canceration of infected cells (Vargas et al., 2019; Olesen et al., 2019; Yu et al., 2018). Although 54.7% of samples were identified as a single HPV genotype, more than one HPV genotype was detected in 214 samples (45.3%). Therefore, it is necessary to reduce the risk of carcinogenesis of infected tissues by increasing the frequency of follow-ups, performing regular pathological examinations for pathological tissues, or strengthening the treatment for high-risk HPV-infected subjects and multiple HPV subtypes (including high-risk subtypes) (Gargiulo et al., 2007). 

Several studies in women have shown that HPV infection is age-specific (Liu et al., 2014; Chen et al., 2017). However, no accurate data were available for men. This study mentions the first example of this age-specific distribution with an unimodal mode. We found that young men under the age of 31 had the highest infection rates. This is probably because they are new to sex, have unsensitized immune systems, and are susceptible to HPV infection. They represented a high-risk group for HPV but generally showed transient infections. HPV infection rates decrease with age but generally remain high between the ages of 31 and 40. The reason may be viral persistence or latent HPV reactivation due to physiological and immunological disturbances during the menopausal transition (Kang et al., 2014; Althof et al., 2009). Therefore, implementing a vaccination program before young people can become infected with HPV may be effective in reducing HPV infection rates. 

Furthermore, when analyzing the frequencies of LR and HR HPV HPV genotypes, our technology had a positive advantage in calculating the concentration of each genotype expressed as protocol (copies/sample). It has been difficult to present the values in a quantitative form to track changes for positive or negative results. Although we have not had the opportunity to compare the quantitative results with the cytological grade, viral copy numbers showed high sensitivity at concentrations as low as 0.1 log (copies/sample) and as high as 13.3 log (copies/sample). While genotype 51, which had the second-highest concentration of copies per sample (5.38 log), was shown to be the most common second type similar to HPV studies in men (Yin et al., 2020; Laura et al., 2019). In the analytical procedure, the amplified product contains both a conserved region and a highly differentiated region facilitating the design of both the generic and type-specific probes respectively. New technologies might add some specific probes to increase the sensitivity for the detection of HPV51. Despite the presence of degenerate base sites, amplification across some HPV genotypes is uneven due to mismatches or low viral loads of specific HPV types. Further studies are planned to measure and define the high levels of infection that may lead to the development of urological tumors. Using this technique, clinical decisions to grade cytological changes may be possible. 

The large sample size and application of a standard and approved commercial HPV diagnostic kit were two important encouraging points of our study which let us compare our findings with others. Overall, it can be concluded that HPV infection is currently at a considerably high level in Iran. Looking at the high risk and oncogenic HPV subtypes frequency in studied samples, especially in younger age groups, a concern could be noticed about HPV-relevant cancers which can be prevented by commercial and approved HPV vaccines.

There remain limitations in the present study. First, the present study was retrospective, not a randomized controlled trial, and no blinding method was established. Therefore, there is still a risk of bias due to causes such as the different severity of the patient’s disease, the different duration of the disease, and the previous use of various drugs. Second, the lack of detailed information on the study population’s geographic, socio-demographic, and behavioral characteristics of the study population prevents assessment of these characteristics on the HPV infection rate. But, the most attractive advantage of the present study is a multi-center clinical study. However, a larger and more representative population is recommended for future research. 

In conclusion, regional data on HPV prevalence and genotype distribution are needed to estimate the impact of vaccines on malignancies and screening programs. Because HPV immunity is genotype-specific, it is important to determine the prevalence of LR and HR HPV in different geographic regions. Most of the previous studies have focused on a woman’s HPV infection in the general population. Few have studied the epidemiology of HPV infection in men in high-risk populations. In Turkey, this is the first comprehensive prevalence study of her HPV genotyping other than her HPV 16 and 18. Moreover, the quantification of the viral genome was an important factor in the decision-making process. We believe that the promotion and spread of bivalent, tetravalent vaccines have reduced the infection rate of HPV types 16 and 18. However, the overall infection rate of HPV continues to increase. HPV vaccination should be encouraged at community and government levels (Sopian et al., 2019), Prevention of malignant diseases still has a long way to go (Yan et al., 2021). Currently, three prophylactic HPV vaccines (bivalent, quadrivalent, and nine-valent) are in use worldwide, but WHO does not make any biased recommendations for these three vaccines (Kazlouskaya et al., 2019). Regarding the value of vaccines, it is not true that more is better, only targeted vaccines provide effective prevention (Lin et al., 2019). Turkey should consider both cost-effectiveness and the type of actual HPV infection when choosing an appropriate vaccine.

## Author Contribution Statement

All authors contributed equally in this study.
